# IL-6, IL-17 and Stat3 are required for auto-inflammatory syndrome development in mouse

**DOI:** 10.1038/s41598-018-34173-5

**Published:** 2018-10-25

**Authors:** Takatsugu Oike, Hiroya Kanagawa, Yuiko Sato, Tami Kobayashi, Hiroko Nakatsukasa, Kana Miyamoto, Satoshi Nakamura, Yosuke Kaneko, Shu Kobayashi, Kengo Harato, Akihiko Yoshimura, Yoichiro Iwakura, Tsutomu Takeuchi, Morio Matsumoto, Masaya Nakamura, Yasuo Niki, Takeshi Miyamoto

**Affiliations:** 1 Department of Orthopedic Surgery, Keio University School of Medicine, 35 Shinano-machi, Shinjuku-ku, Tokyo 160-8582 Japan; 2 Department of Advanced Therapy for Musculoskeletal Disorders, Keio University School of Medicine, 35 Shinano-machi, Shinjuku-ku, Tokyo 160-8582 Japan; 3 Department of Musculoskeletal Reconstruction and Regeneration Surgery, Keio University School of Medicine, 35 Shinano-machi, Shinjuku-ku, Tokyo 160-8582 Japan; 4 Department of Microbiology and Immunology, Keio University School of Medicine, 35 Shinano-machi, Shinjuku-ku, Tokyo 160-8582 Japan; 5 Division of Rheumatology, Department of Internal Medicine, Keio University School of Medicine, 35 Shinano-machi, Shinjuku-ku, Tokyo 160-8582 Japan; 6 Division of Experimental Animal Immunology, Center for Animal Disease Models, Research Institute for Biomedical Sciences, Tokyo University of Science, 2641 Yamazaki, Noda-shi, Chiba 278-8510 Japan

## Abstract

Auto-inflammatory syndrome, a condition clinically distinct from rheumatoid arthritis, is characterized by systemic inflammation in tissues such as major joints, skin, and internal organs. Autonomous innate-immune activation is thought to promote this inflammation, but underlying pathological mechanisms have not been clarified nor are treatment strategies established. Here, we newly established a mouse model in which IL-1 signaling is conditionally activated in adult mice (hIL-1 cTg) and observed phenotypes similar to those seen in auto-inflammatory syndrome patients. In serum of hIL-1 cTg mice, IL-6 and IL-17 levels significantly increased, and signal transducer and activator of transcription 3 (Stat3) was activated in joints. When we crossed hIL-1 cTg with either IL-6- or IL-17-deficient mice or with Stat3 conditional knockout mice, phenotypes seen in hIL-1 cTg mice were significantly ameliorated. Thus, IL-6, IL-17 and Stat3 all represent potential therapeutic targets for this syndrome.

## Introduction

Auto-inflammatory syndrome is marked by systemic inflammation including arthritis, increased white blood cell counts in peripheral blood, and internal organ dysfunction^1,2^. Patients with auto-inflammatory syndrome exhibit major joint dominant arthritis and several extra-articular symptoms distinct from manifestations of rheumatoid arthritis (RA)^3,4^. Historically, TNF receptor-associated periodic syndrome (TRAPS) was first reported by McDermott *et al*. in 1999^5^, leading to our current understanding of auto-inflammatory syndromes. Currently, diseases such as Familial Mediterranean Fever (FMF) and Cryopyrin-associated periodic syndrome are categorized as auto-inflammatory syndromes^2,6^. Most develop in infancy and childhood, but some, including TRAPS, FMF and adult-onset Still’s disease (AOSD), develop in juveniles and adults^3,7,8^. Some auto-inflammatory syndromes emerge from known mutations, although unknown mechanisms still underlie many^2^. Some patients with auto-inflammatory syndrome are treated with colchicine or steroids and others with biologic agents such as anti-IL-6 receptor or anti-IL-1 antibodies^7,9,10^. Nonetheless, a gold standard auto-inflammatory syndrome treatment has not yet been established, as these conditions are rare and pathological mechanisms underlying them are unclear.

Activation of the inflammasome, a protein complex activating inflammatory cytokine expression and apoptosis, reportedly triggers auto-inflammatory syndrome development^11,12^. IL-1 is implicated in auto-inflammatory syndrome pathogenesis^13^, while TNFα, IL-6 and IL-17 are implicated in RA development^14,15^. Animal models such as TNFα-transgenic mice and collagen-induced arthritis models have been established as RA models, leading to better understanding of this condition, while a model of auto-inflammatory syndrome is not yet available. Mouse strains such as DBA1 or Balb/c have been critical for development of arthritis disease models, while the C57BL/6 strain is known to be resistant to arthritis development^16,17^. Given that several mutant mouse lines have been established on a C57BL/6 background, the roles of these candidate genes in arthritis development are likely obscured and remain unknown.

IL-1 overactivation reportedly promotes auto-inflammatory syndrome development in human subjects^12,13,18,19^. Patients with a condition known as Deficiency of IL-1 Receptor Antagonist (DIRA), which is caused by a recessive mutation in the *IL1 RN* gene, exhibit severe arthritis and joint destruction^20^. IL-1ra-deficient or IL-1 overexpressing transgenic mice also reportedly exhibit arthritis development^21–23^. Thus, IL-1 receptor antagonists have been considered useful as treatments for patients with DIRA^20,24,25^.

Here, we newly established an adult-onset auto-inflammatory syndrome transgenic mouse model in which IL-1 signals can be conditionally activated at any age after birth by PolyI-PolyC injection. All adult hIL-1 cTg mice on a C57BL/6 background exhibited major joint dominant arthritis and displayed other symptoms seen in auto-inflammatory syndrome patients, such as increased WBC and splenomegaly. When we crossed IL-1 cTg with either IL-6-, IL-17A/F-deficient or Stat3 conditional knockout mice, we observed significant inhibition of arthritis development. Our study may shed light on the pathogenesis underlying auto-inflammatory syndromes and provide information relevant to treatment of patients with these conditions.

## Materials and Methods

### Mice

We purchased C57BL/6 mice from Sankyo Labo Service (Tokyo, Japan). IL-6 KO and IL-17A/F KO mice were generated previously^26,27^. Stat3 conditional knockout (Stat3 cKO) mice were purchased from Oriental Yeast Co., Ltd (Tokyo, Japan). Mice were kept under specific pathogen-free conditions in animal facilities certified by the Keio University animal care committee.

### Generation of human IL-1α conditional transgenic mice (cTg mice)

A human IL-1α conditional transgenic (hIL-1 cTg) construct was generated by linking the chick actin (CAG) promoter with a *neomycin resistance* (*Neo*) gene plus a poly A sequence, with *Neo-poly A* flanked by floxP sites, followed by the human *IL-1α* gene. That construct was microinjected into fertilized eggs, and eggs were then transplanted into recipient oviducts. Offspring harboring the *hIL-1 cTg* transgene were crossed with Mx Cre transgenic mice to establish Mx Cre/hIL-1 cTg mice, hereafter called hIL-1 cTg mice. hIL-1 cTg mice were further crossed with either IL-6 KO, IL-17 KO or Stat3 cKO mice to yield hIL-1 cTg/IL-6 KO, hIL-1 cTg/IL-17 KO or hIL-1 cTg/Stat3 cKO mice, respectively.

### Induction of human IL-1α in conditional transgenic mice and arthritis analysis

Human IL-1α expression was induced in 8-week-old male human IL-1α conditional transgenic mice (hIL-1 cTg) by injecting 200 µl of a solution containing 250 µg of PolyI-PolyC (Sigma-Aldrich Co., St. Louis, MO, USA) for 3 consecutive days intraperitoneally. Some mice were induced with CD-4-depletive or ISO type control antibody (each 5 mg/kg)^28^, followed by additional PolyI-PolyC injection at 9 and 10 weeks of age. Some hIL-1 cTg mice were not treated with PolyI-PolyC. Arthritis severity was evaluated by measuring the ankle thickness before and after PolyI-PolyC injection at various time points.

### Peripheral blood cell count and Enzyme-Linked Immunosorbent Assay (ELISA) analysis

Peripheral blood was collected from control and hIL-1 cTg mice three weeks after PolyI-PolyC injection. White blood cell, platelet and hemoglobin counts were determined using a Celltac MEK-6450 analyzer (Nihon Kohden, Tokyo, Japan).

Whole cell lysates were prepared from peripheral blood of each mouse using RIPA buffer (1% Tween 20, 0.1% SDS, 150 mM NaCl, 10 mM Tris-HCl (pH 7.4), 0.25 mM phenylmethylsulfonylfluoride, 10 μg/mL aprotinin, 10 μg/mL leupeptin, 1 mM Na3VO4, 5 mM NaF (Sigma-Aldrich Co.)). Sera were obtained from peripheral blood of each mouse, and cytokine levels were analyzed using the Luminex^®^200^TM^ System (Luminex Corporation, Austin, TX, USA). An ELISA assay for human IL-1α in cell lysate and sera was undertaken following the manufacturer’s instructions (R&D systems, Minneapolis, MN, USA).

### Histological arthritis score

Ankle joints were removed from control and hIL-1 cTg mice three weeks after PolyI-PolyC injection, and each sample was stained with Safranin O. Safranin O-positive areas were scored as previously described^29^. Articular cartilage damage was assessed in sagittal sections of ankle joints and graded according to a modified Mankin histologic score for the talus articular side^30^. A total modified Mankin score representing the overall state of cartilage in the joint was calculated for each set of experiments.

### Three-dimensional microcomputed tomography (Micro-CT) analysis

Changes in bony microstructure around the ankles were assessed in mice three weeks after PolyI-PolyC injection using micro-CT with an X-ray micro-CT system (CosmoScan GX; Rigaku Corporation, Tokyo, Japan). Eroded areas as a proportion of total cortical bone area were calculated at ankle joints.

### Histopathological and fluorescent immunohistochemical analysis of joints

Ankle joints were removed from control and hIL-1 cTg mice three weeks after PolyI-PolyC injection, fixed in 10% neutral-buffered formalin and embedded in paraffin, and then tissue blocks were cut into 4-μm sections. Ankles were decalcified in 10% EDTA, pH7.4, before embedding. Hematoxylin and eosin (H&E) or safranin-O staining was performed according to standard procedures. For each fluorescent immunohistochemistry assay, sections were subjected to microwave treatment for 10 min in 10 mM citrate buffer solution (pH 6.0) for antigen retrieval. After blocking with 3% BSA in PBS for 1 h, sections were stained for 6 h with rabbit anti-mouse pSTAT3 (1:100 dilution; Cell Signaling Techniques, Inc.), mouse anti-mouse pSTAT3 (1:100 dilution; Cell Signaling Techniques, Inc.), rabbit anti-mouse IL-6 (1:100 dilution; Abcam), rabbit anti-mouse CD45 (1:100 dilution; Abcam), rabbit anti-mouse COL6A1 (1:100 dilution; Abcam), rabbit anti-mouse COL1A1 (1:100 dilution; Rockland), rabbit anti-mouse Myeloperoxidase (1:100 dilution; Abcam) or rabbit anti-mouse IL-17 (1:100 dilution; Abcam) at 4 °C. After washing in PBS, sections were stained with Alexa Fluor 488-conjugated goat anti-rabbit IgG (1:100 dilution; Invitrogen, for pStat3 in Fig. 2), Alexa Fluor 488-conjugated goat anti-mouse IgG (1:100 dilution; Invitrogen, for pStat3 in Figs S2 and S4) or Alexa Fluor 546-conjugated goat anti-rabbit IgG (1:100 dilution; Invitrogen, for IL-6, CD45, COL1A1, COL6A1, Myeloperoxidase and IL-17) for 1 h at room temperature and observed under a fluorescence microscope (Keyence, Tokyo, Japan). To use Alexa Fluor 488-conjugated goat anti-mouse IgG, specimens were pretreated with 10% normal goat serum in PBS for 1 h.

### Flow cytometeric analysis and sorting

Antibodies purchased from BioLegend and eBioscience were diluted 1:400. For IL-17A intracellular staining, cells were stimulated 4 h in complete medium with PMA (50 ng ml^−1^) and ionomycin (1000 ng ml^−1^; both from Sigma-Aldrich) in the presence of brefeldin A (eBioscience). Surface staining was then performed in the presence of Fc-blocking antibodies (2.4G2), followed by intracellular staining with anti-IL-17A antibodies in IC fixation buffer (#00-8222-49,eBioscience), according to the manufacturer’s instructions. We performed flow cytometry acquisition on a FACS Canto II cytometer (BD Biosciences, San Jose, CA, USA) and analysed data using FlowJo software (Tree Star, Ashland, OR, USA). We sorted mouse CD3-positive, B220-positive, Gr-1-negative/CD11b-positive, Gr-1-positive/CD11b-positive and CD45-ngetaive cells using a FACS Aria II system (BD Biosciences).

### Antibodies for flow cytometry

Fluorophore-conjugated monoclonal anti-mouse CD3ε (FITC, 145-2C11), anti-mouse Gr1 (APC, 1A8), anti-mouse B220 (PerCP-Cy5.5, RA3-6B2), anti-mouse CD11b (PE, M1/70), anti-mouse CD45 (APC-Cy7, 30-F11), anti-mouse TCRβ (FITC, H57-597), anti-mouse TCRγδ (APC, GL3), anti-mouse CD4 (PerCP-Cy5.5, RM4-5), anti-mouse IL17A (PE, 17B7), anti-mouse CD11b (FITC, M1/70), anti-mouse CD11c (PerCP-Cy5.5, N418), anti-mouse CD49b (APC, DX5) were purchased from eBioscience (San Diego, CA, USA), Bio Legend (San Diego, CA, USA) or TONBO Bioscience (San Diego, CA, USA).

### Realtime PCR analysis

Total RNAs were isolated from sorted cells using TRIzol reagent (Invitrogen Corp.), and cDNA was generated using oligo(dT) primers and reverse transcriptase (Wako Pure Chemicals Industries). Quantitative realtime PCR was performed using SYBR Premix ExTaq II reagent and a DICE Thermal cycler (Takara Bio Inc.), according to the manufacturer’s instructions. *β-actin* (*Actb*) expression was analyzed as an internal control. Primers for realtime PCR were as follows.

- *β-actin (mouse)*-forward: 5′-TGAGAGGGAAATCGTGCGTGAC-3′
- *β-actin (mouse)*-reverse: 5′-AAGAAGGAAGGCTGGAAAAGAG-3′
- *IL-1α (human)*-forward: 5′-CTTTTAGCTTCCTGAGCAATGTGA-3′
- *IL-1α (human)*-reverse: 5′-TGGTCTCACTACCTGTGATGGTTT-3′

### Statistical analysis

Results are expressed as means ± s.d. Statistical significance of differences between groups was evaluated using Student’s t-test (*P < 0.05; **P < 0.01; ***P < 0.001; NS, not significant, throughout the paper).

### Study approval

Mice protocols were approved by that committee, and all experiments were carried out based on committee guidelines.

## Results

### Establishment of IL-1 cTg mice

**Figure 1:**
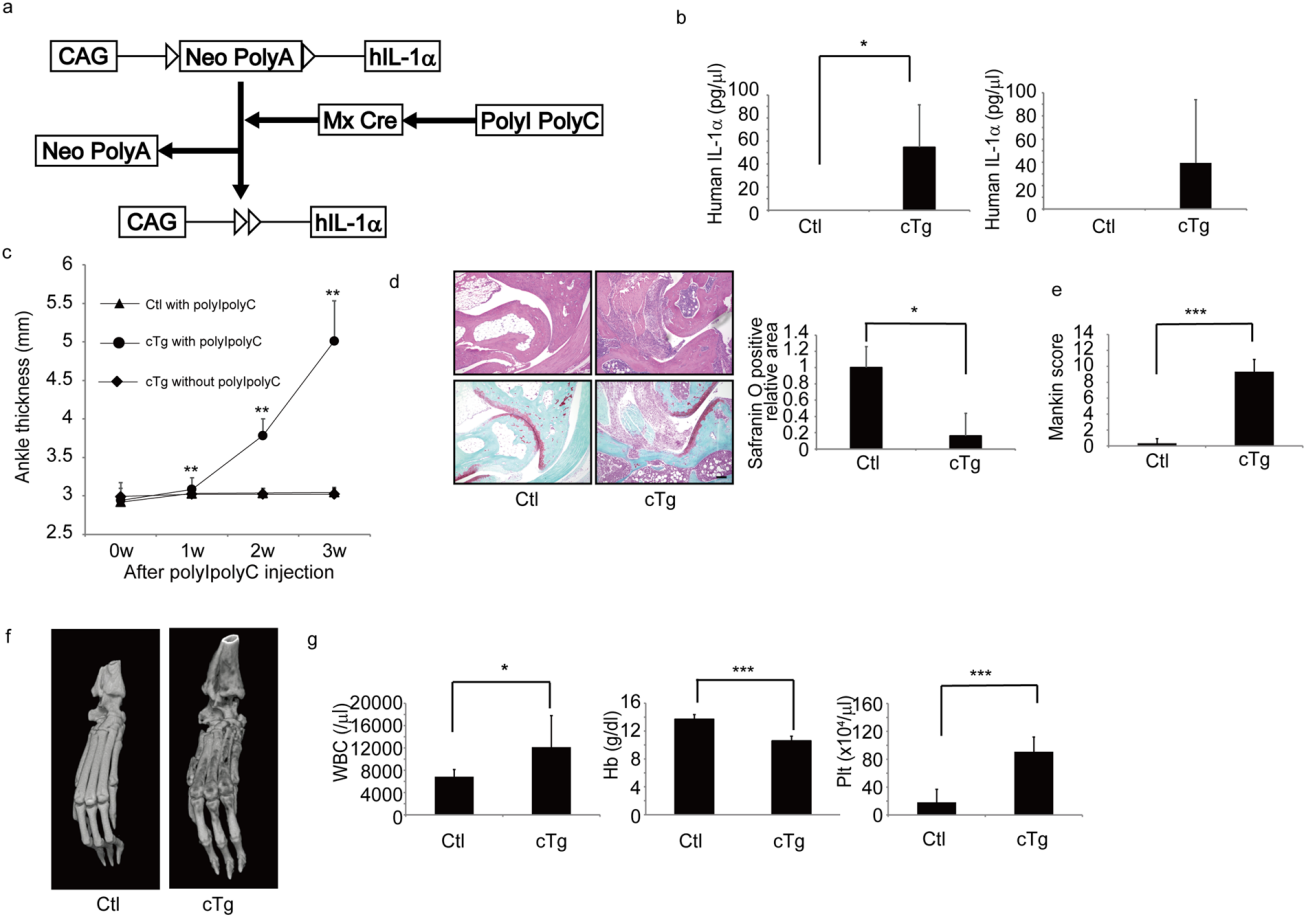
Upregulation of human IL-1α in adult mice promotes major joint dominant arthritis. (**a**) Schematic showing construct used to construct inducible human IL-1α (hIL-1α) conditional transgenic mice. The chick actin promoter (CAG) was linked to a *neomycin resistance* gene (Neo) and a *poly A* sequence, and both were flanked by floxP sites (triangles). The *hIL-1α* gene was then inserted downstream of those sequences to yield hIL-1α transgenic construct. The construct was injected into C57/Bl6 mouse embryo to become hIL-1α transgenic mice, which were crossed with Mx Cre transgenic mice to establish inducible hIL-1α conditional transgenic mice (hIL-1α cTg). PolyI-PolyC injection of these mice activated Cre expression via the Mx promoter, excising the Neo-Poly A sequence and enabling hIL-1α expression driven by the CAG promoter. (**b**–**g**) PolyI-PolyC was injected into eight-week-old control (Ctl) or hIL-1α cTg (cTg) mice, and three weeks after injection, peripheral blood mononuclear cells (left panel) and sera (right panel) were isolated, and hIL-1α protein levels determined by ELISA (b). Data in (b) represent mean human IL-1α (pg/ml) ± SD (left, n = 3 for Ctl; n = 5 for cTg; ^*^P < 0.05; right, n = 3 for Ctl; n = 3 for cTg). Ankle thickness was measured at indicated time points (c). Ankle thickness of cTg mice not treated with PolyI-PolyC is shown in (c). Ankle thickness is shown as mean thickness ± SD (c) (n = 3 for controls treated with PolyI-PolyC, n = 3 for cTg mice treated with PolyI-PolyC, and n = 3 cTg mice not treated with PolyI-PolyC, ^**^P < 0.01). Ankle tissue specimens from Ctl or cTg mice were stained with hematoxylin eosin (HE, left upper panels) or safranin O and methyl green (left lower panels) three weeks after PolyI-PolyC injection when mice were 11 weeks old (d), the safranin O-positive articular cartilage area was scored (d, right panel) (n = 3 for control; n = 3 for cTg mice; ^*^P < 0.05) and Mankin scores were evaluated (e) (n = 3 for control; n = 3 for cTg mice; ^***^P < 0.001). Bar, 100 µm. Bone destruction at ankle joints in Ctl or cTg mice was evaluated by using micro-CT analysis three weeks after PolyI-PolyC injection when mice were 11 weeks old (f). White blood cell (WBC), hemoglobin (Hb) or platelet (Plt) counts were also evaluated (g). Data in (g) represent mean WBC, Hb or Plt counts in peripheral blood ± SD (n = 7 for control; n = 3 for cTg mice; ^*^P < 0.05, ^***^P < 0.001).

First, we created a conditional IL-1 transgenic construct in which the chick actin promoter (*CAG*) was linked to a *neomycin (Neo)-poly A* sequence flanked by flox sites and followed by the human *IL-1α* (*hIL-1α*) sequence (Fig. 1a). At steady state, the CAG promoter activity promotes *Neo* expression terminated by the poly A sequence, blocking induction of hIL-1α expression (Fig. 1a). We injected that construct into C57BL/6 mouse embryos to establish an hIL-1α transgenic line in this strain. We then crossed hIL-1α transgenic mice with Mx Cre mice to yield Mx Cre/hIL-1α transgenic mice, hereafter called hIL-1α conditional transgenic mice (hIL-1α cTg) (Fig. 1a). In hIL-1α cTg mice, injection of PolyI-PolyC activated the Mx promoter and excised the *Neo-poly A* sequence, enabling hIL-1α expression (Fig. 1a).

For all experiments, we injected PolyI-PolyC into eight-week-old hIL-1α cTg or control (Ctl) mice. Three weeks later, we isolated peripheral blood mononuclear cells and determined intracellular hIL-1 levels by ELISA (Fig. 1b). As shown in Fig. 1b, hIL-1α protein was specifically detected in cells or sera from hIL-1α cTg mice, indicating successful establishment of the Tg line. In these conditions, we detected major joint dominant arthritis development in 100% of hIL-1α cTg mice but did not observe arthritis in control mice before or after PolyI-PolyC injection. When we evaluated ankle joint swelling in hIL-1α cTg mice based on ankle thickness, we found that thickness increased significantly in hIL-1α cTg mice after PolyI-PolyC injection (Fig. 1c). Ankle thickness was unchanged in hIL-1α cTg mice in the absence of PolyI-PolyC injection (Fig. 1c). Hematoxylin and eosin (H&E) staining of joint tissue indicated joint destruction and synovitis in hIL-1α cTg mice by three weeks after PolyI-PolyC injection (Fig. 1d). Safranin-O staining of joint tissues also showed synovitis development and loss of articular cartilage in ankle joints of hIL-1α cTg mice (Fig. 1d). As a result, the Mankin score, as determined by histological examination of articular cartilage damage, was significantly higher in hIL-1α cTg than in control mice (Fig. 1e). Micro CT analysis demonstrated severe joint destruction in hIL-1α cTg relative to control mice (Fig. 1f). Peripheral blood tests showed significantly elevated white blood cell (WBC) and platelet (Plt) counts, and hIL-1α cTg mice showed anemia, as evidenced by significantly lower hemoglobin (Hb) levels (Fig. 1g).

We also detected significantly larger spleen, splenomegaly, in hIL-1α cTg compared with control mice (Fig. S1a). Hepatitis and dermatitis were also observed in hIL-1α cTg mice, although hIL-1α cTg mice did not exhibit fever (Fig. S1b,c). hIL-1α cTg mice exhibited thinner and thicker respective subcutaneous fatty and dermal layers than did control mice (Fig. S1d). We also detected infiltration of inflammatory cells into dermal tissues in hIL-1α cTg mice (Fig. S1d) and identified those cells as Myeloperoxigenase (MPO)-positive neutrophils (Fig. S1e). hIL-1α cTg also showed lower body temperature than did control mice (Fig. S1f).

### hIL-1α cTg mice show elevated inflammatory cytokine levels

**Figure 2:**
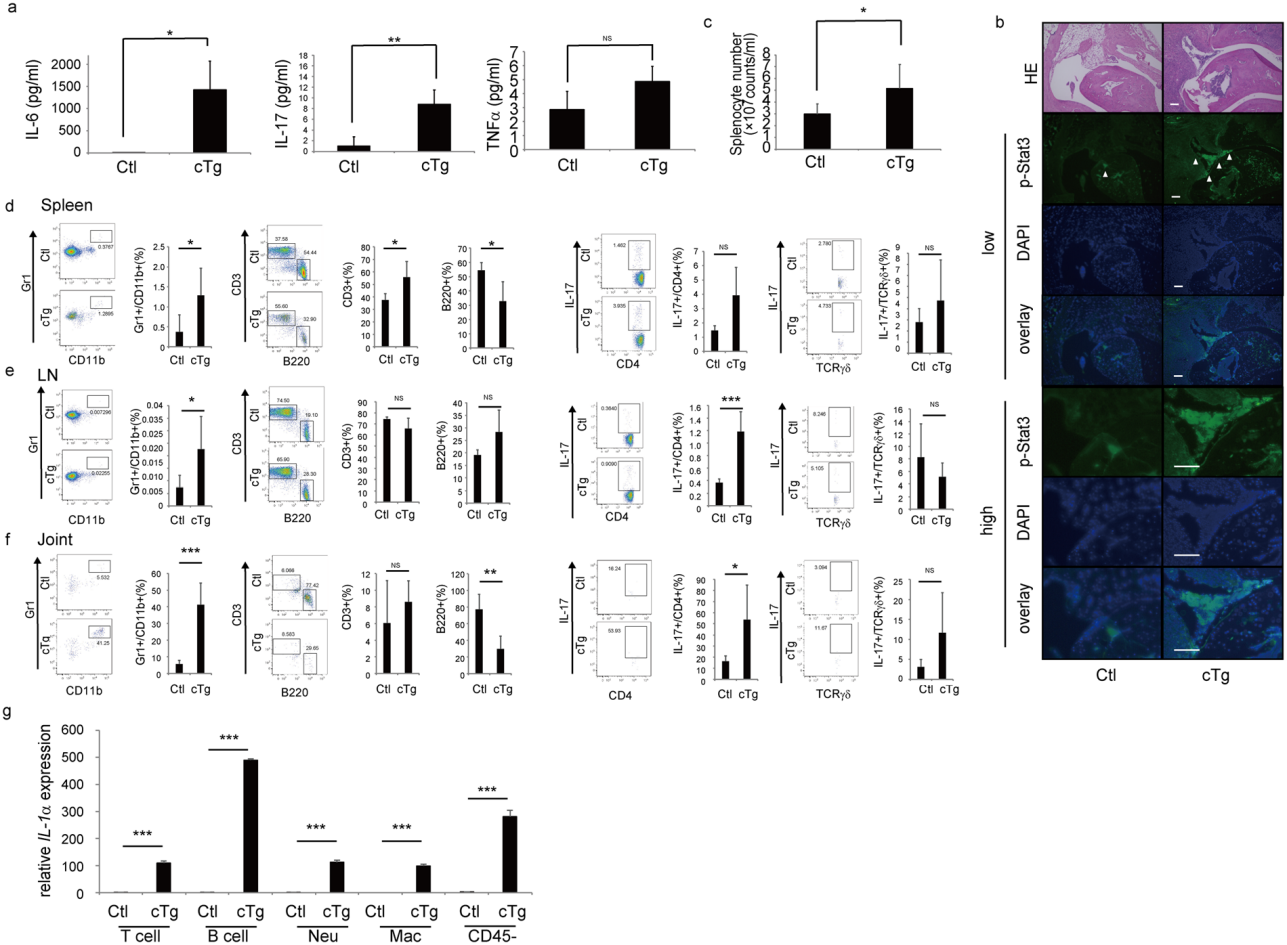
IL-6 and IL-17F/A levels increase and Stat3 is activated in hIL-1α cTg mice. PolyI-PolyC was injected into eight-week-old Ctl or cTg mice. Three weeks later, peripheral blood was collected and ankle joints removed. Levels of cytokines indicated on the y-axis in peripheral blood sera were assessed by ELISA (**a**). Data represent mean levels of indicated cytokines ± SD (n = 3 for Ctl; n = 4 for cTg mice; ^**^P < 0.01, NS not significant). Ankle joint specimens from Ctl or cTg mice were subjected to hematoxylin and eosin (HE) and immunofluorescence staining with an antibody specific for phosphorylated Stat3 (pStat3) Arrowheads indicate pStat3-psotive cells. (**b**) Nuclei were visualized by DAPI. Bar, 100 µm. (**c**) The absolute number of splenocytes in Ctl or cTg mice was determined three weeks after PolyI-PolyC injection. Data represents mean splenocyte number ± SD (n = 6 for Ctl; n = 4 for cTg mice; ^*^P < 0.05). (**d**–**f**) Flow cytometric analysis was undertaken to detect CD11b, Gr1, CD3, B220, CD4, TCRβ, TCRγδ and IL-17 in cells isolated from spleen (d), lymph nodes (LN) (e) and joints (f) from Ctl (upper panels) or cTg (lower panels) mice. TCRβ^+^TCRγδ^−^CD4^+^IL-17^+^ and TCRβ^−^TCRγδ^+^IL-17^+^ cells are shown as IL-17^+^CD4^+^ and IL-17^+^TCRγδ^+^ cells, respectively. Data represent mean frequency (%) of each fraction ± SD (n = 5 for Ctl; n = 4 for cTg mice; ^*^P < 0.05, ^**^P < 0.01, ^***^P < 0.001, NS not significant). (**g**) CD11b− (Mac), Gr1− (Neu), CD3− (T cell) or B220-positive (B cell), or CD45-negative cells (CD45−) were sorted from Ctl or cTg splenocytes, and human *IL-1α* (*hIL-1α*) expression was determined by realtime PCR. Data represent mean indicated gene expression ± SD (n = 3 for Ctl; n = 3 for cTg mice; ^***^P < 0.001).

To assess pathological mechanisms underlying phenotypes seen in hIL-1α cTg mice, we undertook serum ELISA analysis and found that IL-6 and IL-17 levels were significantly higher in hIL-1α cTg than in control mice (Fig. 2a). Immunohistochemical analysis also demonstrated Stat3 activation in ankle joints of hIL-1α cTg mice, an effect not seen in controls (Fig. 2b). We detected activated Stat3 in either Collagen type 6 (Col6)-positive or Collagen type 1 (Col1)-positive synovial or osteoblastic cells, respectively, as well as both CD45-positive or -negative cells in ankle joints of hIL-1α cTg mice (Fig. S2). We also detected IL-6 expression in pStat3-positive cells in ankle joints of hIL-1α cTg mice (Fig. S2). An IL-17-triggered positive feedback loop via IL-6 and Stat3 activation is reported in fibroblasts of arthritis model F759 mice^31^, suggesting that synovial and osteoblasts cell likely produce IL-17 and IL-6.

The absolute number of splenocytes increased significantly in hIL-1α cTg (Fig. 2c), as did the frequency of CD11b/Gr1-positive and CD3-positive cells, while the B220-positive cell population decreased in hIL-1α cTg relative to control mice (Fig. 2d). The frequency of IL-17-positive/CD4-positive cells increased, while the IL-17-positive gamma delta (γδ) T cell population was comparable in control and hIL-1α cTg mouse spleen, lymph nodes and joints (Fig. 2d). Similarly, in hIL-1α cTg mice CD11b/Gr1-positive cells increased in lymph nodes and joints, as did the IL-17-positive/CD4-positive T cell population (Fig. 2e,f). We detected *hIL-1α* mRNA expression in CD11b− (macrophages), Gr1− (neutrophils) CD3− (T cells) or B220-positive (B cells) and in CD45-negative cells in hIL-1α cTg but not control mice (Fig. 2g).

### IL-6 is required for arthritis development in hIL-1α cTg mice

**Figure 3:**
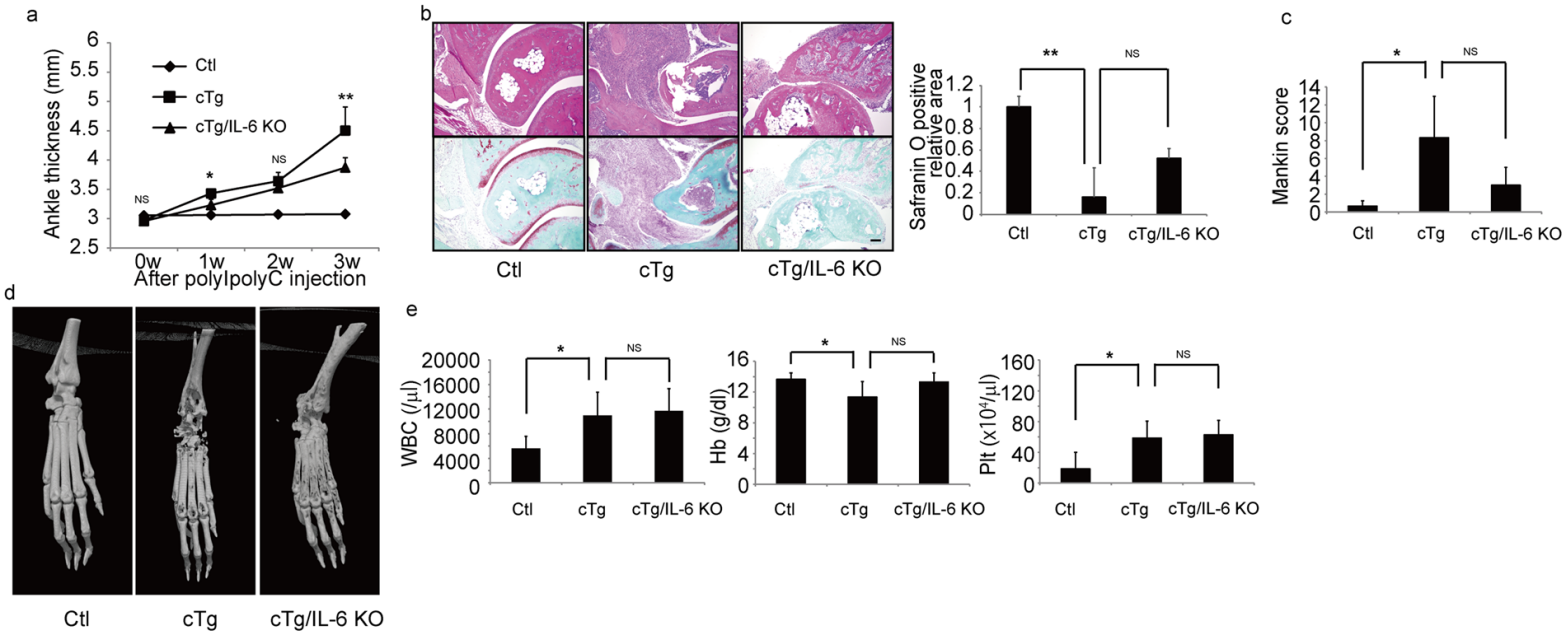
IL-6-deficiency rescues phenotypes seen in hIL-1α cTg mice. hIL-1 cTg (cTg) mice were crossed with IL-6-deficient (IL-6 KO) mice to yield cTg/IL-6 KO mice. PolyI-PolyC was injected into eight-week-old Ctl, cTg or cTg/IL-6 KO mice, and ankle thickness measured at indicated time points (**a**). Data represent mean ankle thickness ± SD (n = 3 for control; n = 3 for cTg mice; n = 8 for cTg+IL-6 KO; ^*^P < 0.05, ^**^P < 0.01, NS not significant, cTg vs cTg/IL-6 KO). Three weeks after PolyI-PolyC injection, ankle joints (**b**–**d**) and peripheral blood (**e**) were collected from each mouse. Ankle tissue specimens from all three genotypes were stained with HE (upper panels) or safranin O and methyl green (lower panels) (**b**), the safranin O-positive articular cartilage area was scored (**b**) and Mankin scores were evaluated (**c**). Data in (**b**,**c**) represent mean safranin O-positive area (**b**) (n = 3 for control; n = 3 for cTg mice; n = 3 for cTg+IL-17 KO mice; ^**^P < 0.01, NS not significant) or Mankin score (**c**) ± SD (n = 3 for control; n = 3 for cTg mice; n = 3 for cTg/IL-6 KO mice; ^*^P < 0.05, NS not significant). Bar, 100 µm. Destruction of ankle bone in Ctl, cTg or cTg/IL-6 KO mice was evaluated by micro-CT (**d**). White blood cell (WBC), hemoglobin (Hb) and platelet (Plt) counts in peripheral blood were determined and are shown as mean indicated parameter ± SD (**e**) (n = 6 for control; n = 3 for cTg mice; n = 8 for cTg+IL-6 KO; ^*^P < 0.05, NS not significant).

Since serum IL-6 levels were significantly elevated in hIL-1α cTg mice (Fig. 2a), we crossed hIL-1α cTg mice with IL-6-deficient mice (IL-6 KO) to yield hIL-1α cTg/IL-6 KO mice (Fig. 3). We then injected PolyI-PolyC into eight-week-old double mutant, hIL-1α cTg/IL-6 KO, and control mice. Increased ankle thickness seen in hIL-1α cTg mice after PolyI-PolyC injection was partially but significantly inhibited by IL-6 deficiency (Fig. 3a). Synovitis and loss of articular cartilage were also inhibited, and the articular cartilage area, as detected by safranin O positivity, was significantly smaller in hIL-1α cTg than control mice, a phenotype rescued by IL-6 deletion (Fig. 3b). Also, the elevated Mankin score seen in hIL-1α cTg mice decreased in hIL-1α cTg/IL-6 mice three weeks after PolyI-PolyC injection, although that difference was not significant (Fig. 3c). Joint destruction, as detected by micro CT, was also antagonized in hIL-1α cTg /IL-6 mice relative to hIL-1α cTg mice (Fig. 3d). However, elevated WBC and platelet counts, and reduced hemoglobin seen in hIL-1α cTg mice were not altered by IL-6 deficiency (Fig. 3e).

### IL-17 functions in arthritis development in hIL-1α cTg mice

**Figure 4:**
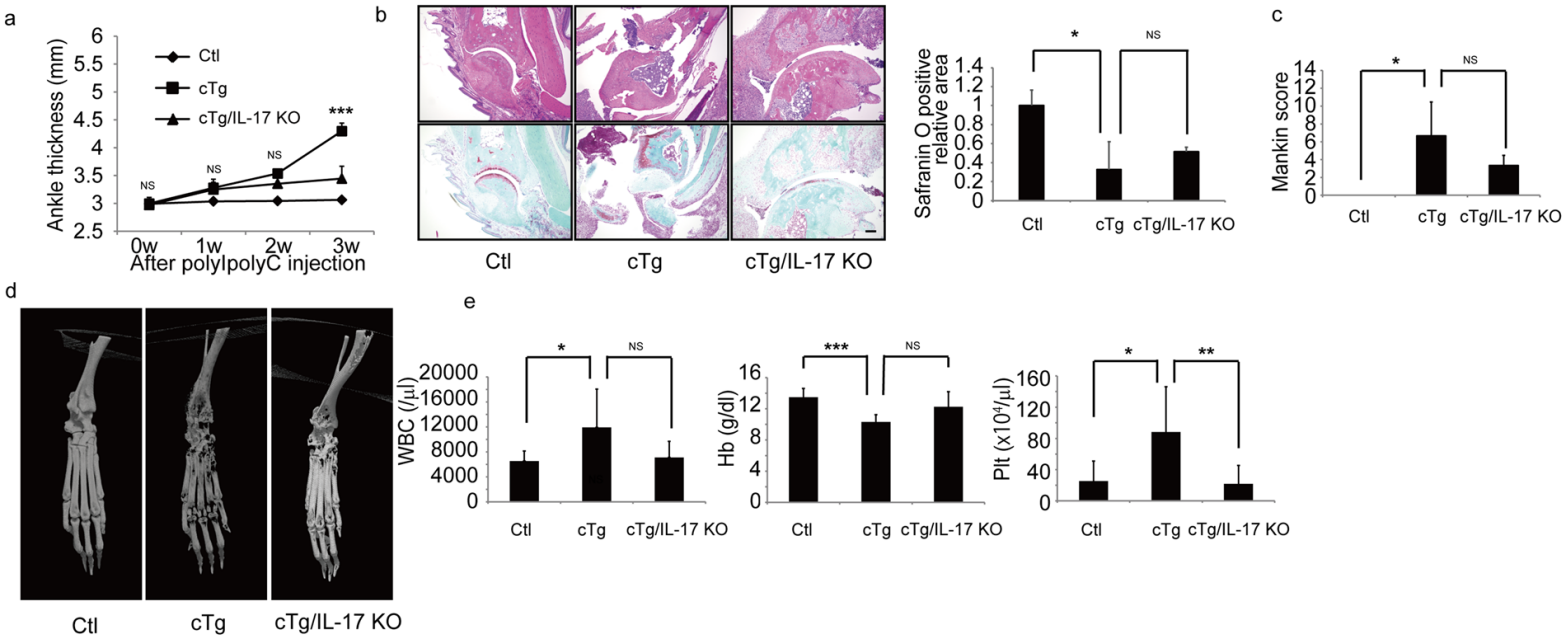
IL-17 deletion blocks hIL-1α cTg phenotypes. hIL-1α cTg (cTg) mice were crossed with IL-17A/F-deficient (IL-17 KO) mice to create cTg/IL-17 KO mice. PolyI-PolyC was injected into eight-week-old Ctl, cTg or cTg/IL-17 KO mice, and ankle thickness evaluated at indicated time points (**a**). Data represent mean ankle thickness ± SD (n = 3 for control; n = 3 for cTg mice; n = 9 for cTg/IL-17 KO; ^***^P < 0.001, NS not significant, cTg vs cTg + IL-17 KO). Three weeks after PolyI-PolyC injection, ankle joints (**b–d**) and peripheral blood (**e**) were isolated, and ankle tissue specimens from all three genotypes were stained with HE (upper panels) or safranin O and methyl green (lower panels) (**b**). The Safranin O-positive articular cartilage area (**b**) and Mankin scores were evaluated (**c**), and shown as mean safranin O-positive area (**b**) (n = 3 for control; n = 3 for cTg mice; n = 3 for cTg+IL-17 KO mice; ^*^P < 0.05, NS not significant) or Mankin score (**c**) ± SD (n = 3 for control; n = 3 for cTg mice; n = 3 for cTg+IL-17 KO mice; ^*^P < 0.05, NS not significant). Bar, 100 µm. Destruction of ankle bone in Ctl, cTg or cTg/IL-17 KO mice was evaluated by micro-CT (**d**). White blood cell (WBC), hemoglobin (Hb) and platelet (Plt) counts in peripheral blood were analyzed and are shown as mean WBC, Hb or Plt ± SD (**e**) (n = 8 for control; n = 5 for cTg mice; n = 9 for cTg+IL-17 KO; ^*^P < 0.05, ^**^P < 0.01, ^***^P < 0.001, NS not significant).

We next undertook a similar cross of hIL-1α cTg mice with IL-17A/F-deficient (IL-17 KO) mice to evaluate potential involvement of the cytokine IL-17 in hIL-1α cTg phenotypes. hIL-1α cTg/IL-17 KO mice showed improvement similar to that shown by hIL-1α cTg/IL-6 mice in terms of arthritis development (Fig. 4). Increased ankle thickness in hIL-1α cTg mice was significantly inhibited in hIL-1α cTg/IL-17 KO mice (Fig. 4a). Synovitis and articular cartilage loss was also inhibited by IL-17A/F-deficiency in hIL-1α cTg mice (Fig. 4b). Although not significant, increases in the Mankin score seen in hIL-1α cTg mice were diminished in hIL-1α cTg/IL-17 KO mice (Fig. 4c). Bone erosion was also inhibited (Fig. 4d), and elevated platelet counts were significantly inhibited in hIL-1α cTg/IL-17 KO relative to hIL-1α cTg mice (Fig. 4e). Moreover, although not significant, both elevation in WBC and reduction in hemoglobin levels appeared to be partially rescued in IL-17-deficient hIL-1α cTg mice (Fig. 4e).

### *Stat3* deletion inhibits arthritis development in hIL-1α cTg mice

**Figure 5:**
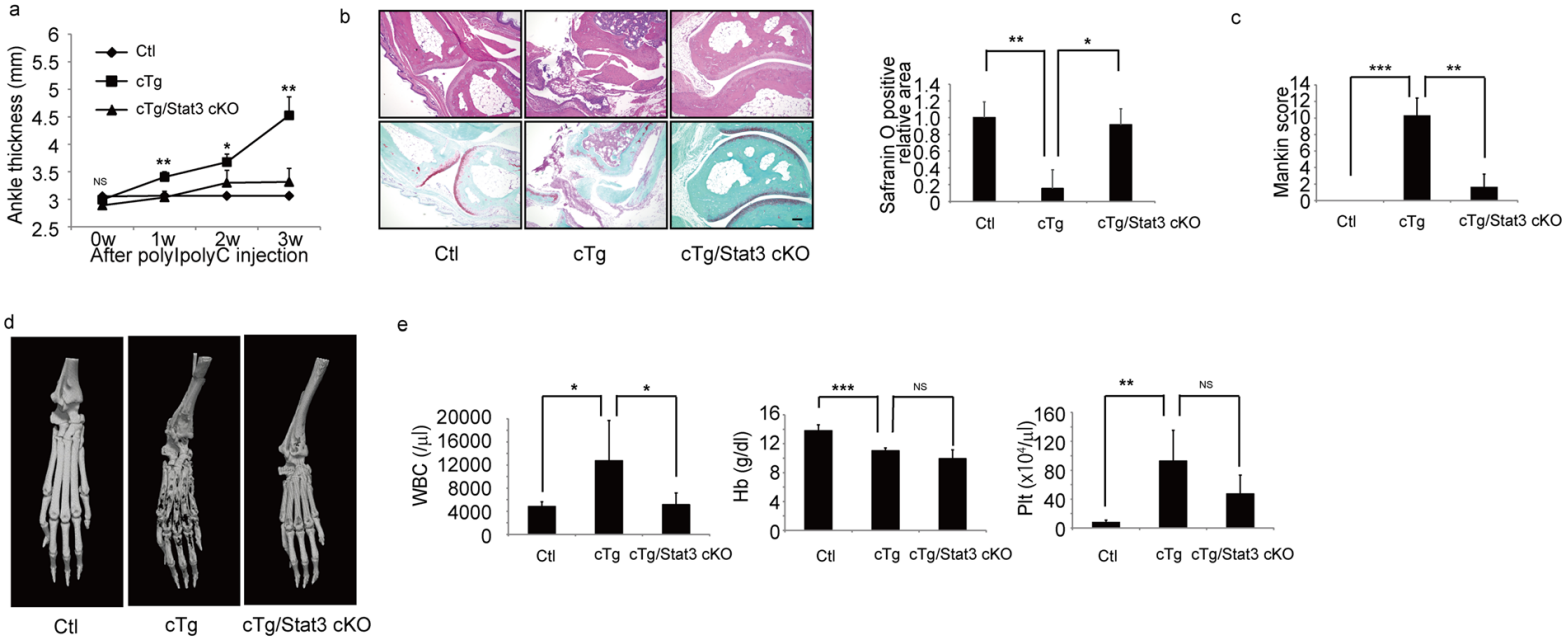
Stat3 deletion rescues hIL-1α cTg phenotypes. hIL-1α cTg **(**cTg) mice were crossed with Stat3 floxed (Stat3 cKO) mice to yield cTg/Stat3 cKO mice. PolyI-PolyC was injected into eight-week-old Ctl, cTg or cTg/Stat3 cKO mice, and ankle thickness evaluated at indicated time points (**a**). Data represent mean ankle thickness ± SD (n = 3 for control; n = 3 for cTg mice; n = 5 for cTg/Stat3 cKO; ^**^P < 0.01, ^*^P < 0.05, NS not significant, cTg vs cTg/Stat3 cKO). Three weeks after PolyI-PolyC injection, ankle joints (**b**–**d**) and peripheral blood (**e**) were collected. Ankle tissue specimens from all three genotypes were stained with HE (upper panels) or safranin O and methyl green (lower panels) (**b**), and the safranin O-positive articular cartilage area (**b**) and Mankin scores were evaluated (**c**). Data in (**b**,**c**) represent mean safranin O-positive area (**b**) (n = 3 for control; n = 3 for cTg mice; n = 4 for cTg + Stat3 cKO mice; ^*^P < 0.05, ^**^P < 0.01) or Mankin score (**c**) ± SD (n = 5 for control; n = 3 for cTg mice; n = 3 for cTg + Stat3 cKO mice; ^**^P < 0.01, ^***^P < 0.001). Bar, 100 µm. Destruction of ankle bone from Ctl, cTg or cTg + Stat3 cKO mice was evaluated using micro-CT (**d**). White blood cell (WBC), hemoglobin (Hb) and platelet (Plt) counts in peripheral blood were scored (**e**). Data represent mean WBC, Hb or Plt ± SD (n = 5 for control; n = 4 for cTg mice; n = 5 for cTg/Stat3 cKO; ^***^P < 0.001, ^**^P < 0.01, ^*^P < 0.05, NS not significant).

Finally, we asked whether Stat3 activation played a role in joint phenotypes seen in hIL-1α cTg mice (Fig. 5). To do so, we crossed *Stat3*^*flox/flox*^ (Stat3 cKO) mice with hIL-1α cTg mice to yield hIL-1α cTg/Stat3 cKO mice. Injection of PolyI-PolyC into hIL-1α cTg/Stat3 cKO mice resulted in Stat3 excision and concomitant elevation in IL-1 levels. Moreover, joint swelling, as assessed by ankle thickness, was significantly inhibited in hIL-1α cTg/Stat3 cKO mice (Fig. 5a). Pannus infiltrate in joints and loss of articular cartilage were also inhibited (Fig. 5b,c), as was elevation of the Mankin score seen in hIL-1α cTg mice in hIL-1α cTg/Stat3 cKO mice (Fig. 5d). Furthermore, increased WBC counts seen in hIL-1α cTg mice were significantly rescued in hIL-1α cTg/Stat3 cKO mice (Fig. 5e).

## Discussion

Auto-inflammatory syndrome(s) are marked by systemic inflammation; those syndromes include diseases such as TRAPS, FMF and AOSD, most of them rare diseases. Patients with these syndromes exhibit major joint dominant arthritis and other systemic inflammatory symptoms, and thus are clinically distinct from RA patients. At present, treatment protocols for RA include administration of disease-modifying anti-rheumatic drugs (DMARDs) followed by biologics. However, since pathological mechanisms underlying auto-inflammatory syndromes are not fully characterized, and the diseases are rare, standard protocols to treat these conditions have not been established. It is currently thought that activation of inflammatory cytokine expression underlies these syndromes^32,33^. Here, we show that mice in which IL-1 signaling is upregulated after birth exhibit symptoms similar to those seen in auto-inflammatory syndrome patients, such as major joint dominant arthritis and splenomegaly. Indeed, administration of CD4-depleting antibody, which effectively inhibits arthritis development in an RA model^34^, to hIL-1α cTg mice did not inhibit arthritis development (Fig. S3). This outcome is likely due to the fact that IL-17 and IL-6 are also expressed in joints of cTg mice (Fig. S4). IL-6 and IL-17 expression in cTg mouse joints was Stat3-dependent, as expression of both decreased in joint tissue of cTg/Stat3 cKO animals (Fig. S4). We demonstrate that, although phenotypes were induced by activated IL-1 signaling, targeting of either IL-6, IL-17 or Stat3 ameliorated those symptoms, suggesting that all of these factors warrant attention as potential therapeutic targets for the syndrome. Indeed, bone erosion was significantly induced by hIL-1α in cTg mice, but that phenotype was significantly blocked in mice lacking either IL-17 or Stat3 (Fig. S5).

Pathological mechanisms underlying RA versus auto-inflammatory syndromes differ in the following ways. Elevated TNFα and/or IL-6 levels promote minor joint dominant arthritis, and activation of T cells such as TH17 cells is required for RA pathogenesis^35–37^. Stat3 is also reportedly required for arthritis development in RA^37–39^. By contrast, inflammasome activation followed by expression of inflammatory cytokines by macrophages^40^ reportedly promotes pathogenesis of auto-inflammatory syndromes^41–43^. However, inflammasome-independent auto-inflammatory syndromes have also been reported^44,45^; thus not all mechanisms underlying auto-inflammatory syndromes have been defined^46^. Inflammasomes were activated in auto-inflammatory syndrome patients, who also exhibited conversion of pro-IL-1β to an active form to secret by caspase 1 activity^47,48^. Inflammasomes are reportedly activated by activities such as macrophage activation^49,50^; however, molecular mechanisms underlying these processes remain unclear. In our model, serum IL-1β levels increased in hIL-1α cTg mice (Fig. S6), suggesting that IL-1α stimulates inflammasome activation, and our model recapitulates, at least in part, human auto-inflammatory syndrome. Here, we show that activation of IL-1 signaling promotes major joint dominant arthritis, and that upregulation of either of IL-6, IL-17 or Stat3, which also function in RA, was required for arthritic phenotypes seen in hIL-1 cTg mice.

At present, we do not know how activated IL-1 signals trigger IL-6 and IL-17 expression and Stat3 activation. Previously, we demonstrated that IL-1 signals promote IL-6 expression followed by activation of a Stat3-dependent auto-amplification loop responsible for IL-6 production^38^. IL-6 is known to promote IL-17 expression^51^. Furthermore, the IL-17-triggered positive feedback loop between IL-17 and IL-6 and its downstream effector Stat3 reportedly promotes arthritis development in gp130 mutant F759 mice^31^. In our mouse model, serum IL-17 and IL-6 levels were significantly elevated, Stat3 was activated in joints following IL-1 induction, and arthritis development was significantly blocked by deletion of IL-17, IL-6 or Stat3, suggesting that the IL-17-mediated positive feedback loop of inflammatory cytokines is induced by IL-1. Why arthritis occurs in minor versus major joints is a subject for future investigation.

Animal models have helped investigators understand the pathogenesis of many diseases. Both RA and auto-inflammatory syndromes develop after birth, and adult-onset animal models relevant to RA, such as CIA and AIA, are available; moreover, the function of IL-17 and Stat3 in RA development has been demonstrated using animal models^22,29,35,38^. By contrast, there are currently no adult-onset animal models available to study auto-inflammatory syndromes. Here, we established an adult-onset auto-inflammatory syndrome model using the Cre-loxP system to activate IL-1 signaling, which promoted systemic inflammation in adults as evidenced by inflammasome activation. We were able to establish this model on a C57BL/6 background, a strain considered resistant to arthritis development. We then crossed this model with C57BL/6 mice harboring various mutations in genes encoding cytokines and a transcription factor in order to identify potential effectors of IL-1 signaling. That analysis identified 3 factors, IL-6, IL-17, and Stat3, that may serve as therapeutic targets in treatment of auto-inflammatory syndromes. IL-6 is reportedly a therapeutic target in several auto-inflammatory diseases^52–54^; however, IL-17 and Stat3 have not been previously identified as therapeutic targets in these conditions.

Among auto-inflammatory syndromes, AOSD shows greatest similarity to our hIL-1 cTg mice, although our mice do not exhibit all phenotypes seen in patients with AOSD. Still’s disease is also an arthritis disease first described by Still in 1897^55^ and currently known as systemic juvenile idiopathic arthritis^56^. In 1971, Bywaters described 14 children with pediatric Still’s disease resembling AOSD^57^. AOSD is diagnosed by 5 criteria, which must include at least two major ones. Major criteria include: arthralgia for more than two weeks, intermittent fever for longer than a week, typical rash, and a WBC greater than 10,000. Minor criteria include: sore throat, lymphadenopathy and/or splenomegaly, abnormal liver function tests (LFT), and anti-nuclear antibody (ANA)- and rheumatoid factor (RF)-negative status. Exclusion criteria include infection, malignancies and rheumatic diseases. A limitation of our study is that differences exist between human auto inflammatory diseases and our models. For example, we did not detect fever in our mouse models (Fig. S1f). Also, serum ferritin levels are reportedly frequently elevated in AOSD patients^58^, an effect we also did not observe in our models. We cannot yet explain differences between the human syndrome and phenotypes seen in animal models. Nonetheless, patients with AOSD have been treated with non-steroidal anti-inflammatory drugs (NSAIDs), corticosteroids, methotrexate and cyclosporine, and more recently with anti-IL-1 receptor or anti-IL-6 receptor antibodies^33,59^; however, consistent strategies useful to treat AOSD are not yet established. Our study provides valuable information relevant to possible therapeutic options for auto-inflammatory syndromes.

## Electronic supplementary material

Supplementary Figures and Legends
