# Supplementary material for: IL-6, IL-17 and Stat3 are required for auto-inflammatory syndrome development in mouse
Source: Sci Rep. 2018 Oct 25;8:15783. doi: 10.1038/s41598-018-34173-5 (PMC6202393; doi:10.1038/s41598-018-34173-5)
Supplement: Supplementary file 1 — Supplementary Figures and Legends [file 41598_2018_34173_MOESM1_ESM.pdf]

IL-6, IL-17 and Stat3 are required for auto-inflammatory syndrome development in mouse

Takatsugu Oike[1], Hiroya Kanagawa[1], Yuiko Sato[1,2], Tami Kobayashi[1,3], Hiroko Nakatsukasa[4], Kana Miyamoto[1], Satoshi Nakamura[1], Yosuke Kaneko[1], Shu Kobayashi[1], Kengo Harato[1], Akihiko Yoshimura[4], Yoichiro Iwakura[6], Tsutomu Takeuchi[5], Morio Matsumoto[1], Masaya Nakamura[1], Yasuo Niki[1] and Takeshi Miyamoto[1,2]

[1]Department of Orthopedic Surgery, [2]Department of Advanced Therapy for Musculoskeletal Disorders, [3]Department of Musculoskeletal Reconstruction and Regeneration Surgery, [4]Department of Microbiology and Immunology, [5]Division of Rheumatology, Department of Internal Medicine, Keio University School of Medicine, 35 Shinano-machi, Shinjuku-ku, Tokyo 160-8582, Japan, [6]Division of Experimental Animal Immunology, Center for Animal Disease Models, Research Institute for Biomedical Sciences, Tokyo University of Science, 2641 Yamazaki, Noda-shi, Chiba 278-8510, Japan

Supplemental Figure Legends

Figure S1. Spleen, skin and liver phenotypes in IL-1α cTg mice.

(a) Spleen weight was evaluated at three weeks after PolyI-PolyC injection when mice were 11 weeks old (n =11 for control (Ctl), n =8 for cTg mice, ***P < 0.001). (b) Liver tissue specimens from Ctl or cTg mice were stained with hematoxylin eosin three weeks after PolyI-PolyC injection when mice were 11 weeks old. (c) Appearance of Ctl or cTg mice three weeks after PolyI-PolyC injection. (d) Dermal tissues were stained with HE and observed under a microscope. Arrow (F) and arrow (D) indicate respective widths of fatty and dermal layers. Bar, 100 μm. (e) Dermal tissues were stained with rabbit anti-MPO antibody followed by Alexa546-conjugated anti-rabbit Igs antibody. Nuclei were stained with DAPI, and tissues were observed under a fluorescence microscope. Bar, 100 μm. (f) Rectal temperature was evaluated at 8 (before PolyI-PolyC injection) and 11 weeks of age (after PolyI-PolyC injection) (n =12 for control, n =8 for cTg mice, *P < 0.05).

Figure S2. pStat3 was detected in either Col6-positive or Col1-positive, or both CD45-positive and -negative cells in ankle joints of hIL-1α cTg mice.

PolyI-PolyC was injected into eight-week-old Ctl or cTg mice. Three weeks later ankle joints removed. Ankle joint specimens from Ctl or cTg mice were subjected to immunofluorescence staining with an antibodies specific for phosphorylated Stat3 (pStat3), CD45, Col6, Col1 or IL-6. Arrowheads indicate pStat3/IL-6 doubly-positive

cells.

Figure S3. CD4-depletion does not effectively inhibit arthritis development in IL-1α cTg mice.

PolyI-PolyC was injected into eight-week-old control (Ctl) or hIL-1α cTg (cTg) mice, with or without anti-CD4 (GK1.5) or ISO type control (ISO) antibody, and ankle thickness measured at indicated time points. Data represent mean ankle thickness ± SD (n=3 for control; n=3 for cTg mice with anti-CD4 antibody; n=3 for cTg mice with IOS type control antibody; NS not significant, cTg/ISO type vs cTg/anti-CD4).

Figure S4. IL-17 and IL-6 expression in synovial cells and subchondral bone is Stat3-dependent.

PolyI-PolyC was injected intraperitoneally into eight-week-old control (Ctl), hIL-1α cTg (cTg) or cTg/Stat3 cKO mice, and ankle joints removed three weeks later. Joint specimens from Ctl, cTg or cTg/Stat3 cKO mice were then subjected to immunofluorescence staining with antibodies specific for phosphorylated Stat3 (pStat3), IL-17 or IL-6. Nuclei were stained with DAPI, and tissues were observed under a fluorescence microscope. Bar, 100 μm.

Figure S5. Bone erosion is significantly induced in IL-1α cTg mice but blocked by IL-17 or Stat3 deletion.

PolyI-PolyC was injected into eight-week-old control (Ctl) or into hIL-1α cTg (cTg),

cTg/IL-6 KO, cTg/IL-17 KO or cTg/Stat3 cKO mice. Three weeks later, ankle joints were removed, micro-CT analysis was performed, and the eroded area per cortical bone area was scored. Data represent mean eroded area/cortical bone area ± SD (n=3 each; *P < 0.05, NS not significant).

Figure S6. Serum mouse IL-1β levels increase in hIL-1α cTg mice.

PolyI-PolyC was injected into eight-week-old control (Ctl) or hIL-1α cTg (cTg) mice, and three weeks later, sera were collected and mouse IL-1β protein levels determined by ELISA. Data represent mean IL-1β (pg/ml) ± SD (n=3 for Ctl; n=3 for cTg; *P < 0.05).

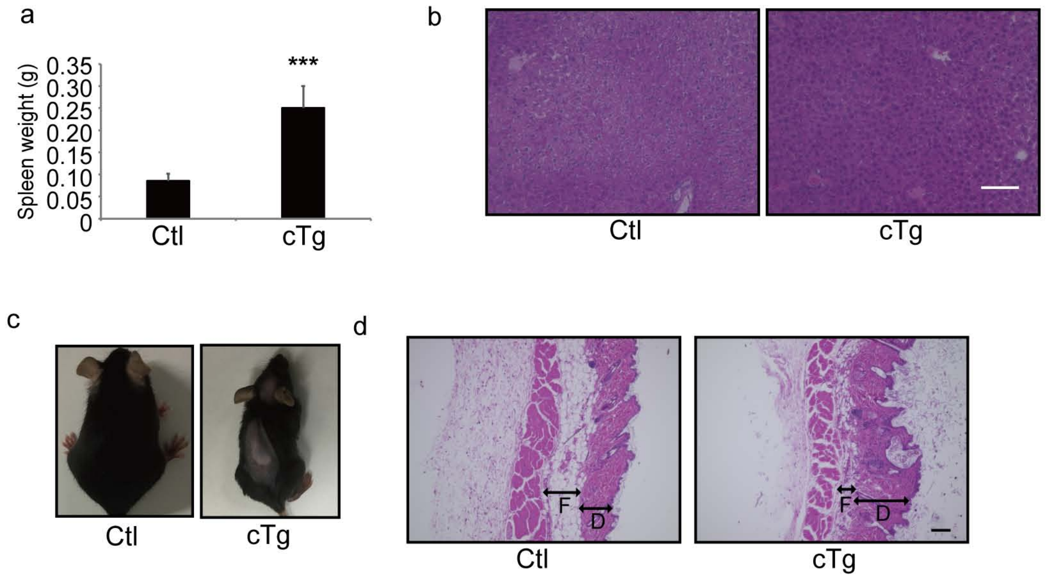

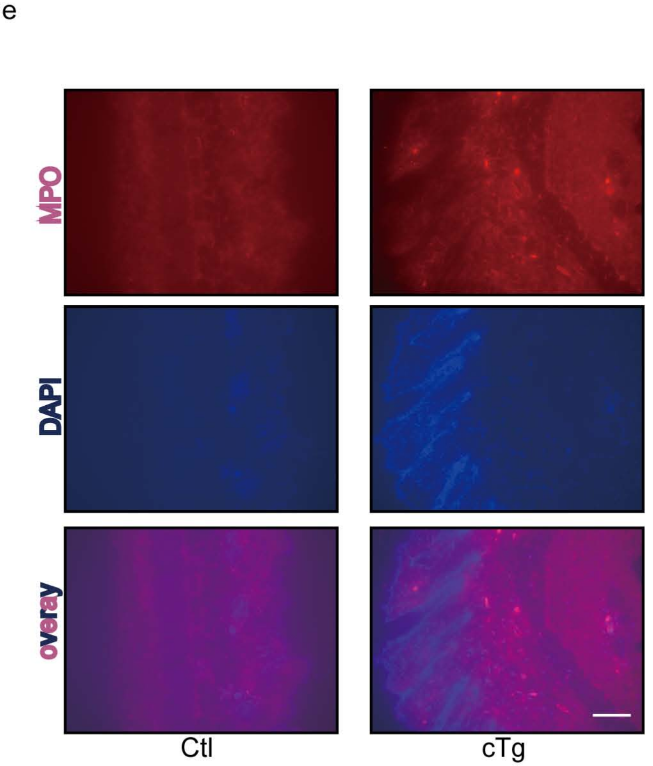

f

Week	8	11
Rectal temperature(℃)		
Control	36.7±0.5	37.7±0.8
cTg	37.0±0.6	36.7±1.0 *

Figure S1.Oike T.et al.

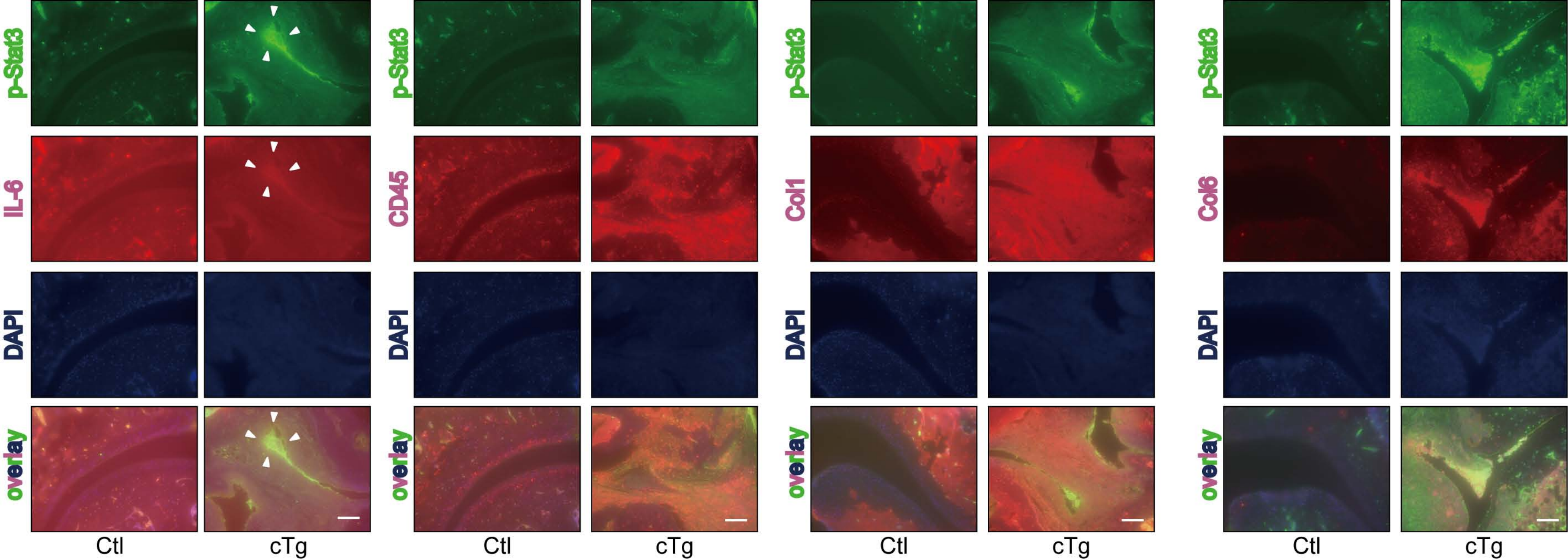

Figure S2.Oike T.et al.

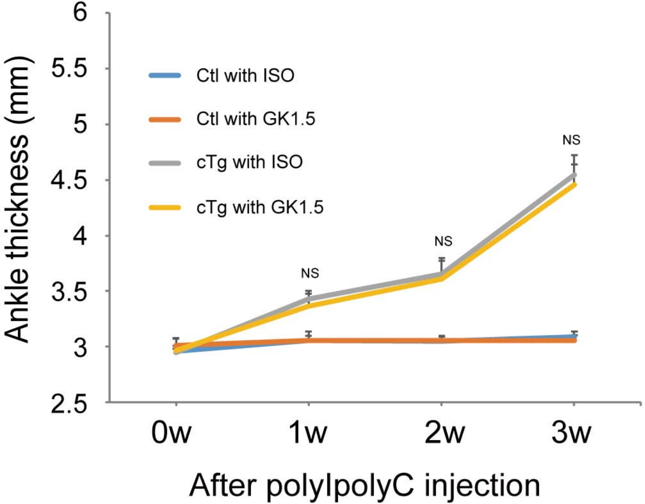

Figure S3.Oike T.et al.

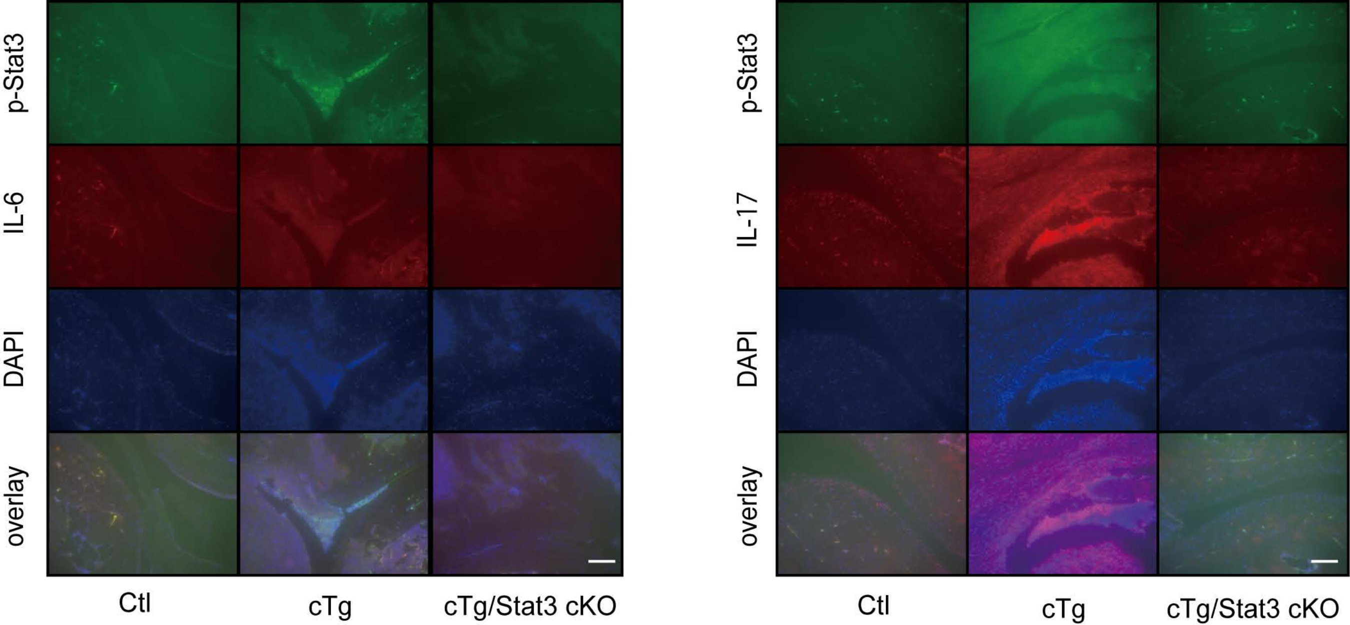

Figure S4.Oike T.et al.

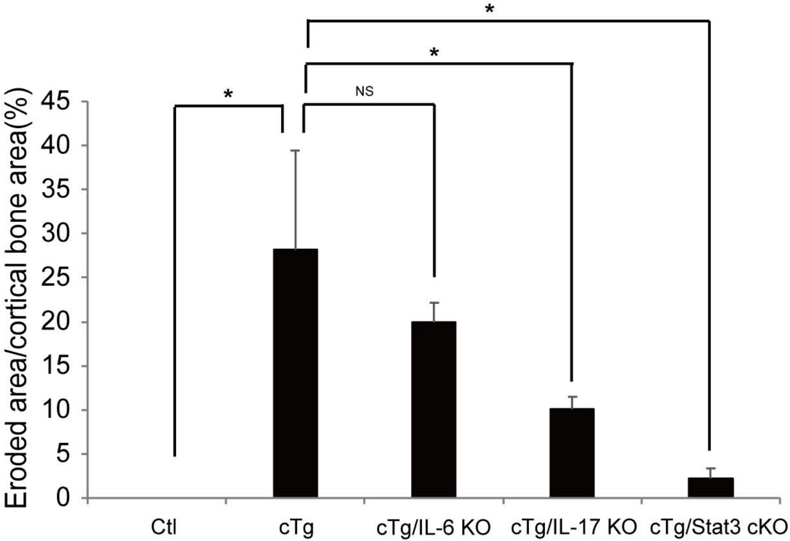

Figure S5.Oike T.et al.

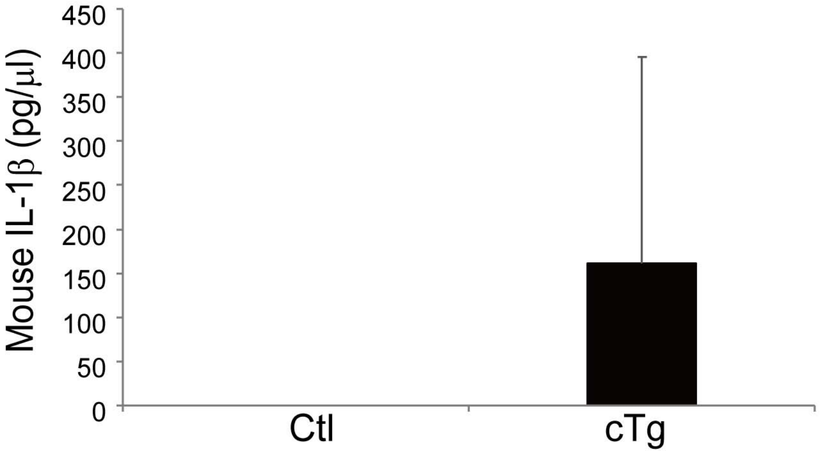

Figure S6.Oike T.et al.
